# A phase-to-intensity strategy of angular velocity measurement based on photonic orbital angular momentum

**DOI:** 10.1515/nanoph-2021-0461

**Published:** 2021-09-28

**Authors:** Duo Deng, Hua Zhao, Jincheng Ni, Yan Li, Cheng-Wei Qiu

**Affiliations:** Department of Optoelectronics Science, Harbin Institute of Technology, Weihai, 264209, China; School of Physics, Harbin Institute of Technology, Harbin, 150001, China; Department of Electrical and Computer Engineering, National University of Singapore, Singapore, 117583, Singapore

**Keywords:** optical diffraction, optical vortex, orbital angular momentum, remote sensing

## Abstract

Recently, orbital angular momentum (OAM) has been adopted to measure the shape of static objects and the translation motion of moving objects in optical remote sensing. Most of these studies rely on measuring the intensity variation of OAM beams. However, the OAM intensity does not change with the rotation of the spinning object, but its phase changes. The phase variation is proved to be proportional to the object’s angular velocity. Since a rotating object will cause the OAM phase dependent on time, the OAM phase needs to be measured instantaneously, to support the OAM-based angular velocity measurement. In this work, we report a scheme to measure the angular velocity of a spinning object using a photonic OAM phase spectrum. A phase-to-intensity strategy is implemented to enable the real-time multi-OAM phase measurement, in which the phase can be determined with the intensities of four focal spots in a two-dimensional array generated by a phase-only spatial light modulator. The experimental results show that the average error of the measured angular velocity could be under 2.45% by detecting the phase of two OAM modes. This OAM-based angular velocity detection method provides a complementary approach to characterize the rotational Doppler effect, especially for slow angular motion.

## 1 Introduction

Lights carrying orbital angular momentum (OAM) have attracted tremendous attention due to their omnipresent applications in quantum optics [1], [2], [3], optical manipulations [4], detection [5], imaging [6], and communications [7], [8], [9]. OAM beams have a helical wavefront described by exp (*ilϕ*), where *l* is the topological charge (TC) and *ϕ* is azimuthal angle [10]. The theoretical infinity of TC gives OAM beams the capacity to be used as information carriers, which greatly improves the transmission capacity of the communication system [8]. The helical phase structure of the OAM beam is stable and constant in the direction of propagation [11], which enables the OAM beam to be used as a new probe beam in the field of remote sensing. Recently, remote sensing technology using OAM has been investigated in a plurality of contexts including detecting object’s parameters [12, 13], rectilinear motion [14, 15], angular velocity [16], [17], [18], [19], [20], [21], [22], chirality [23], defect [24], and refraction index [25]. Furthermore, such technologies show their superior advantages, which have the potential to provide higher sensitivity than conventional Gaussian beam-based technologies [23, 24].

In these investigations, a new type of Doppler effect, named the rotational Doppler effect is the main method applied in probing the angular velocity of spinning objects [16]. It is demonstrated that this effect causes a modulation frequency shift on the scattered light, which is proportional to the angular velocity Ω of the spinning object and the mode of the OAM beam. Therefore, the angular velocity can be deduced by detecting the frequency shift of the scattered OAM light from a spinning object with an optically rough surface. This method can be applied in both the light wave [17] and the radio frequency regime [19]. However, whether in the laboratory [19] or outdoor conditions [22], when using the rotational Doppler effect to measure the angular velocity of a spinning object, complex devices and a significant number of measurements and computations are required for the frequency measurement. Since the frequency shift caused by the rotational Doppler effect is proportional to the object’s angular velocity, in order to ensure the frequency shift can be measured, this scheme is more available for measuring objects rotating at high speed rather than low speed [18]. Thus, the angular velocity of rotating objects measured by the rotational Doppler effect is often greater than 10^2^ (rad/s) [16], [17], [18], [19], [20], [21] (see Supplementary Table S1). Furthermore, high-order OAM beams should be used to enlarge the frequency shift. But the beam quality decreases with the increment of the mode of the vortex beam [26], which may restrict the applications of the rotational Doppler effect as the scattered light is too weak to be collected. In addition, when measuring the parameters of a static object with vortex beams, Xie et al. [12] observed that the orientation of the object is closely related to the slope between different OAM modes in the OAM phase spectrum. This discovery shows the potential application of the OAM phase spectrum in remote sensing measurement. However, the slope will fluctuate sharply with the error of the OAM phase measurement and the noninstantaneous measurement of the OAM phase limits its application prospect in the field of OAM-based remote sensing. Consequently, measurements of the angular velocity via photonic OAM are not discussed fully.

In this paper, we theoretically proposed and experimentally confirmed a phase-to-intensity strategy of angular velocity remote sensing by using photonic OAM phase spectrum, which is different from the well-known rotational Doppler effect. We proved that when a beam passes through a rotating object, the phase variation of the OAM state in the truncated beam is linearly related to the angular velocity of the object, time, and the OAM mode. Based on that, the angular velocity of the rotating object could be calculated by measuring the OAM phase variation over some time. Therefore, the instantaneous measurement of the OAM phase is essential when the OAM phase spectrum is used to measure the angular velocity of an object. To this end, we proposed a real-time multi-OAM phase measurement method, in which a phase-only mask was designed to generate a two-dimensional phase-to-intensity measurement array (2DPIMA). The relative phase of a single OAM mode could be deduced instantaneously by detecting the intensities of four spots in this array. To reduce the measurement error, the phase spectra of two OAM modes were employed together to calculate the average angular velocity. As a proof-of-concept experiment, a laser with 532 nm wavelength was used to measure the angular velocity of the spinning object and the average experimental error of angular velocity measurement could be under 2.45% using two low-order OAM modes. Compared with the well-known rotational Doppler effect which employs high-order vortex mode and is more suitable for measuring high-speed spinning objects, OAM-based angular velocity detection could break the limitation of OAM mode and supply the low-speed measurement range. This simple sensing method, which shows the potential of using the OAM phase spectrum to measure the speed of moving objects, may find wide applications in OAM-based remote sensing.

## 2 OAM phase spectrum variation caused by object motion

**Figure 1:**
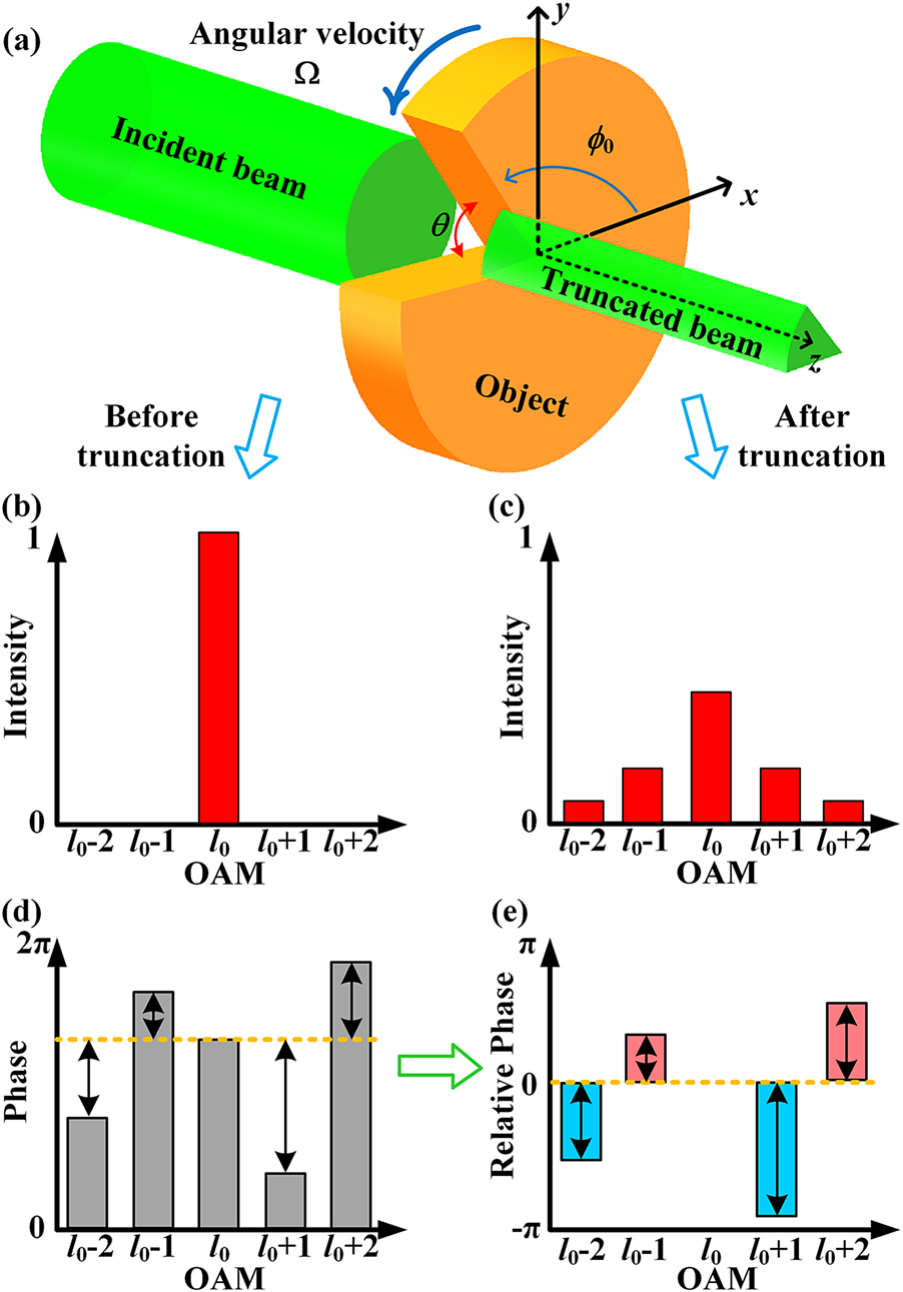
(a) Schematic of the OAM beam passing through a spinning object whose opening angle is *θ* with angular velocity Ω. (b) OAM intensity spectrum before truncation, and (c) after truncation. (d) OAM phase spectrum of the truncated beam, and its corresponding (e) relative phase spectrum.

As Figure 1(a) shown, an incident OAM beam with arbitrary eigenmode *l*_0_ is illuminated onto a spinning object and truncated by the slit of the object. The truncated beam passes through the slit, leading to the discrete OAM spectrum. Specifically, diffraction of the OAM beam happened after the slit and generated extra concomitant OAM states *l*_*n*_ (*l*_*n*_ ≠ *l*_0_) on the truncated beam. This phenomenon could be explained by the theory of OAM angular diffraction [27]. As shown in Figure 1(b) and (c), the OAM intensity spectrum is changed when the beam is truncated by the object. Due to the change of the modes and intensities of OAM beams, the OAM intensity spectrum of the truncated beam has attracted the most attention, and it has been proved that the OAM intensity spectrum is closely related to the size of the object’s slit. However, the OAM intensity spectrum does not change with the rotation of the object (see Supplementary Note 1). Instead of the OAM intensity spectrum, we focus on the OAM phase spectrum of the truncated beam in this work.

When the object spins, the rotation of the slit of the object will cause the variation of the OAM phase spectrum in the truncated beam. In the diffraction field, the complex amplitude of the *l*-th OAM state at the time *t* could be described as [28],

(1) $$A_{l,z}\left(t\right)=\int_{0}^{R}A\left(r\right)\;dr\int_{\phi_{0}+\Omega t}^{\phi_{0}+\theta +\Omega t}\exp\;\left(il_{0}\phi \right)\exp\;\left(i\varphi_{0}\right)\exp\;\left(ikz\right)\exp\;\left(-il\phi \right)d\phi \text{ ,}$$

where *R* is the radius of the incident beam. The amplitude and phase of the incident OAM beam are *A*(*r*) and *φ*_0_, respectively. The angular velocity of the object is Ω. *θ* is the opening angle of the object’s slit. *ϕ*_0_ is the azimuth angle at the initial time. *r* and *ϕ* are the normalized polar coordinates on the receiving plane. *k* = 2*π*/*λ* is the wavenumber, with *λ* is the vacuum wavelength. *z* is the propagation distance. The phase spectrum in Eq. (1) is rewritten as,

(2) $$\varphi_{l,z}\left(t\right)=\left(l_{0}-l\right)\left(\phi_{0}+\Omega t\right)+\frac{\theta }{2}\left(l_{0}-l\right)+\varphi_{0}+kz\text{ .}$$

From Eq. (2), we could see that the phase spectrum includes the incident OAM state *l*_0_ and concomitant OAM states *l*_*n*_, and their phases are different as shown in Figure 1(d). Furthermore, the phase spectrum will change with the propagation of the beam. To eliminate the influence of propagation distance on the phase spectrum, we define the relative phase

(3) $$\Phi_{l}\left(t\right)=\varphi_{l,z}\left(t\right)-\varphi_{l_{0},z}\left(t\right)=\left(l_{0}-l\right)\left(\phi_{0}+\Omega t\right)+\frac{\theta }{2}\left(l_{0}-l\right)\text{ ,}$$

which could be used to indiscriminately describe the OAM phase spectrum on the *z*-axis in the diffraction field. Figure 1(e) shows that the phase of OAM state *l*_0_ is 0 in the relative phase spectrum. For other concomitant OAM states *l*_*n*_, the phase of each OAM state changes with the rotation of the object due to the first factor in Eq. (3). In general, the phase variation of *l*-th OAM in Δ*t* could be described as,

(4) $$\Delta \Phi_{l}=\Phi_{l}\left(t_{0}+\Delta t\right)-\Phi_{l}\left(t_{0}\right)=\left(l_{0}-l\right)\Omega \Delta t\text{ ,}$$

where $\Phi_{l}\;\left(t_{0}\right)$ is the relative phase of the *l*-th OAM state at the initial time *t*_0_ and $\Phi_{l}\;\left(t_{0}+\Delta t\right)$ is the relative phase of the *l*-th OAM state at the time *t*_0_ + Δ*t*. According to Eq. (4), we can see that the rotation of the object will cause the change of OAM phase spectrum in the truncated beam, in which the phase variation linearly responds to the angular velocity of the rotating object, time, and the OAM mode difference. Thus, the angular velocity of the spinning object can be calculated,

(5) $$\Omega =\frac{\Delta \Phi_{l}}{\left(l_{0}-l\right)\Delta t},l\neq l_{0}\text{ .}$$

## 3 Real-time multi-OAM phase measurement

Based on Eq. (5), the determination of angular velocity needs to measure the phase variation of concomitant OAM state *l*_*n*_ in Δ*t*. To this end, the measurement of the OAM phase spectrum needs to be realized. The principle of phase measurement is based on modal decomposition [12, 29], in which four transmittance functions *T*_1_, *T*_2_, *T*_3_, and *T*_4_ are employed to calculate the intermodal phase difference between coaxial propagating OAM beams with TCs *m* and *n*,

(6) $$\begin{array}{l} T_{1}=\left[\exp\;\left(im\phi \right)+\exp\;\left(in\phi \right)\right]/\sqrt{2}\\ T_{2}=\left[\exp\;\left(im\phi \right)+\exp\;\left(in\phi \right)\exp\;\left(i\pi /2\right)\right]/\sqrt{2}\\ T_{3}=\left[\exp\;\left(im\phi \right)+\exp\;\left(in\phi \right)\exp\;\left(i\pi \right)\right]/\sqrt{2}\\ T_{4}=\left[\exp\;\left(im\phi \right)+\exp\;\left(in\phi \right)\exp\;\left(i3\pi /2\right)\right]/\sqrt{2}. \end{array}$$

The intermodal phase difference between OAM states *m* and *n* could be calculated by detecting the power of the corresponding spots *I*_1_, *I*_2_, *I*_3_, and *I*_4_ generated with the transmittance functions *T*_1_, *T*_2_, *T*_3_, and *T*_4_,

(7) $$\Delta \varphi_{m,n}=\varphi_{m}-\varphi_{n}=a\;\tan\;\left(\frac{I_{1}-I_{3}}{I_{2}-I_{4}}\right).$$

Thus, the relative phase of the *l*-th OAM state could be calculated $\Phi_{l}=\Delta \varphi_{l,l_{0}}$ .

**Figure 2:**
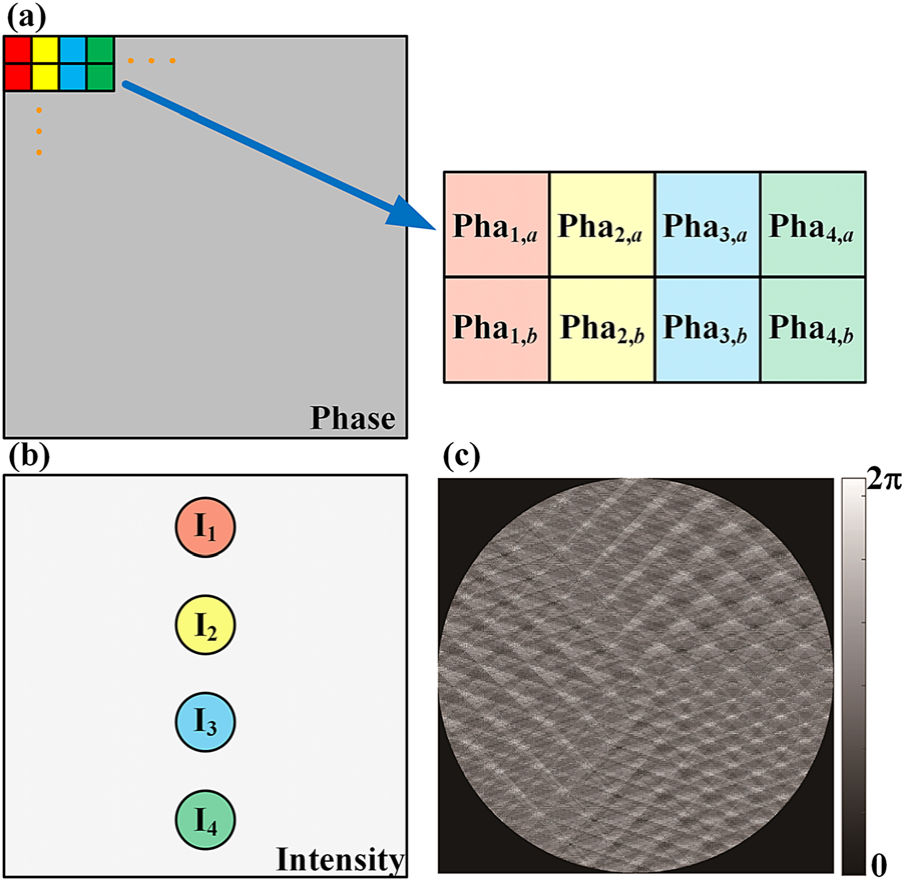
Real-time multi-OAM phase measurement method. (a) Schematic of the phase filling principle of the hybrid phase plate. (b) Two-dimensional phase-to-intensity measurement array. (c) The hybrid phase plate is used to generate an eight-spot 2DPIMA that can measure the real-time relative phase of two OAM states *l* = 1 and *l* = 2.

According to Eq. (3), we can see that the OAM phase spectrum changes with time, so the phase measurement needs to be completed instantaneously. Thus, a real-time multi-OAM phase measurement method is necessary. The key of this phase-to-intensity method is to integrate the four transmittance functions in Eq. (6) into a hybrid phase plate that can generate spots *I*_1_, *I*_2_, *I*_3_, and *I*_4_ in one 2DPIMA. That ensures those intensities can be measured simultaneously. The hybrid phase plate is calculated based on the pixel checkboard method [30]. The principle of phase filling is shown in Figure 2(a), the whole phase plane is divided into many single-pixel lattices, which agrees with the pixel distribution feature of the spatial light modulator (SLM). For single relative phase detection, every eight adjacent pixels are used to form a basis set, which is used to generate spots *I*_1_, *I*_2_, *I*_3_, and *I*_4_, respectively. The transmittance functions in Eq. (6) are rewritten into a phase-only mask, in which the phase of these pixels could be expressed as,

(8) $$\begin{array}{l} {\mathrm{Pha}}_{1,a}=m\phi \\ {\mathrm{Pha}}_{1,b}=n\phi \\ {\mathrm{Pha}}_{2,a}=m\phi \\ {\mathrm{Pha}}_{2,b}=n\phi +\pi /2\\ {\mathrm{Pha}}_{3,a}=m\phi \\ {\mathrm{Pha}}_{3,b}=n\phi +\pi \\ {\mathrm{Pha}}_{4,a}=m\phi \\ {\mathrm{Pha}}_{4,b}=n\phi +3\pi /2\text{ .} \end{array}$$

The phase masks Pha_*x*,*a*_ and Pha_*x*,*b*_ in Eq. (8) are equivalent to the transmittance function *T*_*x*_ in Eq. (6), which can be used to generate one spot whose energy is *I*_*x*_ (*x* = 1, 2, 3 and 4). In addition, these four spots are placed in the identical focal plane. As shown in Figure 2(b), pixels filled with Pha_1,*a*_ and Pha_1,*b*_ are attached with the same position information to ensure that the generated focus *I*_1_ is located at the point *P*_1_ in the 2D plane. Similarly, spots *I*_2_, *I*_3_, and *I*_4_ are generated with phase masks Pha_2,*a*_ and Pha_2,*b*_, Pha_3,*a*_, and Pha_3,*b*_, Pha_4,*a*_, and Pha_4,*b*_, which contain different position information of *P*_2_, *P*_3_, and *P*_4_, respectively. Since the four spots *P*_1_, *P*_2_, *P*_3_, and *P*_4_ are generated in one plane at the same time, the energies *I*_1_, *I*_2_, *I*_3_, and *I*_4_ could be measured simultaneously and the phase measurement could be achieved instantaneously. Furthermore, more foci could be placed in one 2DPIMA to achieve the real-time multi-OAM phase measurement. When the phase plate is evenly filled with two basis sets (16 pixels), it can be used to generate an eight-spot 2DPIMA and measure the relative phase of two OAM modes. As shown in Figure 2(c), it is a hybrid phase plate that can be used to measure the relative phase of *l* = 1 and *l* = 2 instantaneously.

## 4 Measuring angular velocity with OAM phase spectra

**Figure 3:**
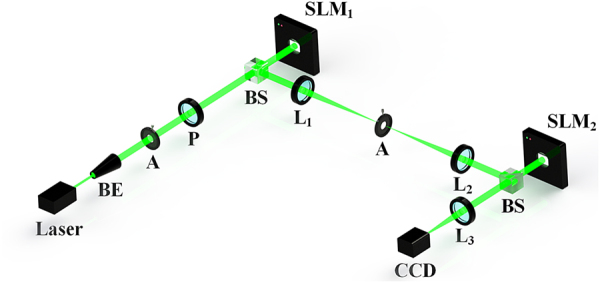
Diagram of the experimental setup. A Gaussian beam generated by the laser is expanded and collimated by a beam expander (BE). Then the beam passes through an aperture (*A*) and a polarizer (*P*), which are used to improve the beam quality and adjust intensity. The well-prepared beam passing through the beam splitter is modulated by the spatial light modulator (SLM_1_). The truncated beam reflected by SLM_1_ is separated by a 4*f* system consisted of lenses (*L*_1_ and *L*_2_, *f *= 200 mm) and an aperture. Then the truncated beam is modulated by the SLM_2_ after reflecting from a beam splitter. After that, a lens *L*_3_ (*f *= 150 mm) is used to focus the beam to a 2DPIMA, where a charge-coupled device (CCD) measures its intensity profile.

The optical setup of the proof-of-concept experiment is depicted in Figure 3. A fundamental mode of the laser beam (*l*_0_ = 0) at the wavelength of 532 nm was collimated to illuminate the SLM_1_. (The OAM state of the incident beam will not influence the OAM angular diffraction. For convenience, a Gaussian beam with *l*_0_ = 0 is employed as the incident beam in this work.) The hologram on SLM_1_ was used to emulate the spinning object frame by frame [31] (see Supplementary Note 2). The Gaussian beam was truncated by the slit of the object and converted into a truncated beam with OAM modes. A 4*f* system with an aperture located at the confocal plane was employed to pick up the first-order diffracted beam. Then the truncated beam was modulated by the SLM_2_, in which a hybrid phase plate as shown in Figure 2(c) was uploaded. Finally, this beam reflected by SLM_2_ was focused by a lens to generate an eight-spot 2DPIMA. The OAM phase spectra of *l* = 1 and *l* = 2 could be calculated instantaneously by the intensities of 2DPIMA’s foci, which were captured by a charge-couple device (CCD) camera (see Supplementary Notes 3 and 4).

**Figure 4:**
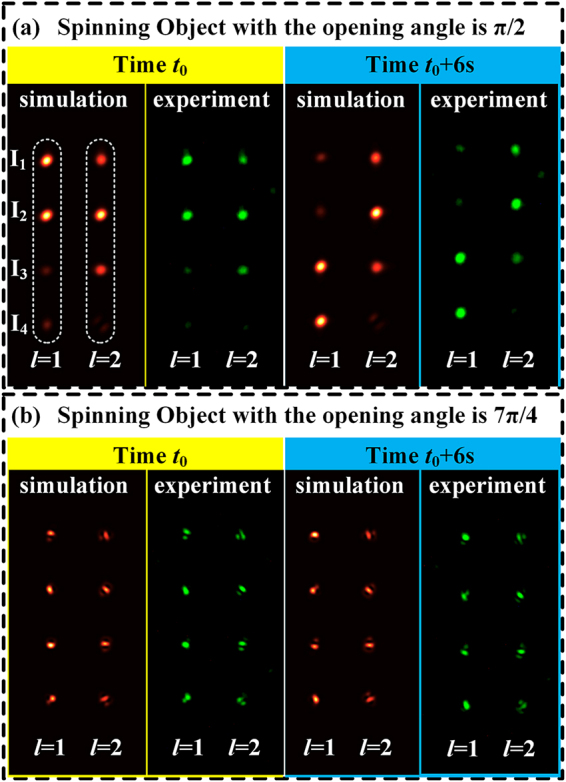
Two-dimensional phase-to-intensity measurement array. (a) Simulation and experimental results of the object with opening angle is *π*/2 measured at *t*_0_, and *t*_0_ + 6 s. (b) Simulation and experimental results of the object with opening angle is 7*π*/4 measured at *t*_0_, and *t*_0_ + 6 s. The angular velocity of both objects was *π*/6 rad/s (counterclockwise rotation). The relative phase of the OAM state could be calculated in real-time by the energy of four foci *I*_1_, *I*_2_, *I*_3_, *I*_4_.

In the experiments, the angular velocities of two spinning objects were measured by OAM phase spectra of two OAM states *l* = 1 and *l* = 2. The opening angle of these two objects is *π*/2 and 7*π*/4, respectively. Firstly, the angular velocity of both objects was set to *π*/6 rad/s (counterclockwise rotation). During the experiments, the diffraction field was captured per second, totally of 12 times. The first and seventh measurements of the 2DPIMA are shown in Figure 4. In each 2DPIMA, the left four collinear spots and the right four collinear spots are used to calculate the real-time relative phase of OAM states *l* = 1 and *l* = 2, respectively. Among them, the results of the simulation and experiment of the first object are shown in Figure 4(a), which were measured at the time *t*_0_ and *t*_0_+6 s (see Supplementary Figure S1). Similarly, Figure 4(b) shows the results of the simulation and experiment of the first and seventh measurements of the second object. Through comparison, we can see that the simulation results are consistent with the experimental results. After that, we increased the angular velocity of both objects to *π*/4 (rad/s) and remeasured the OAM phase spectra.

**Figure 5:**
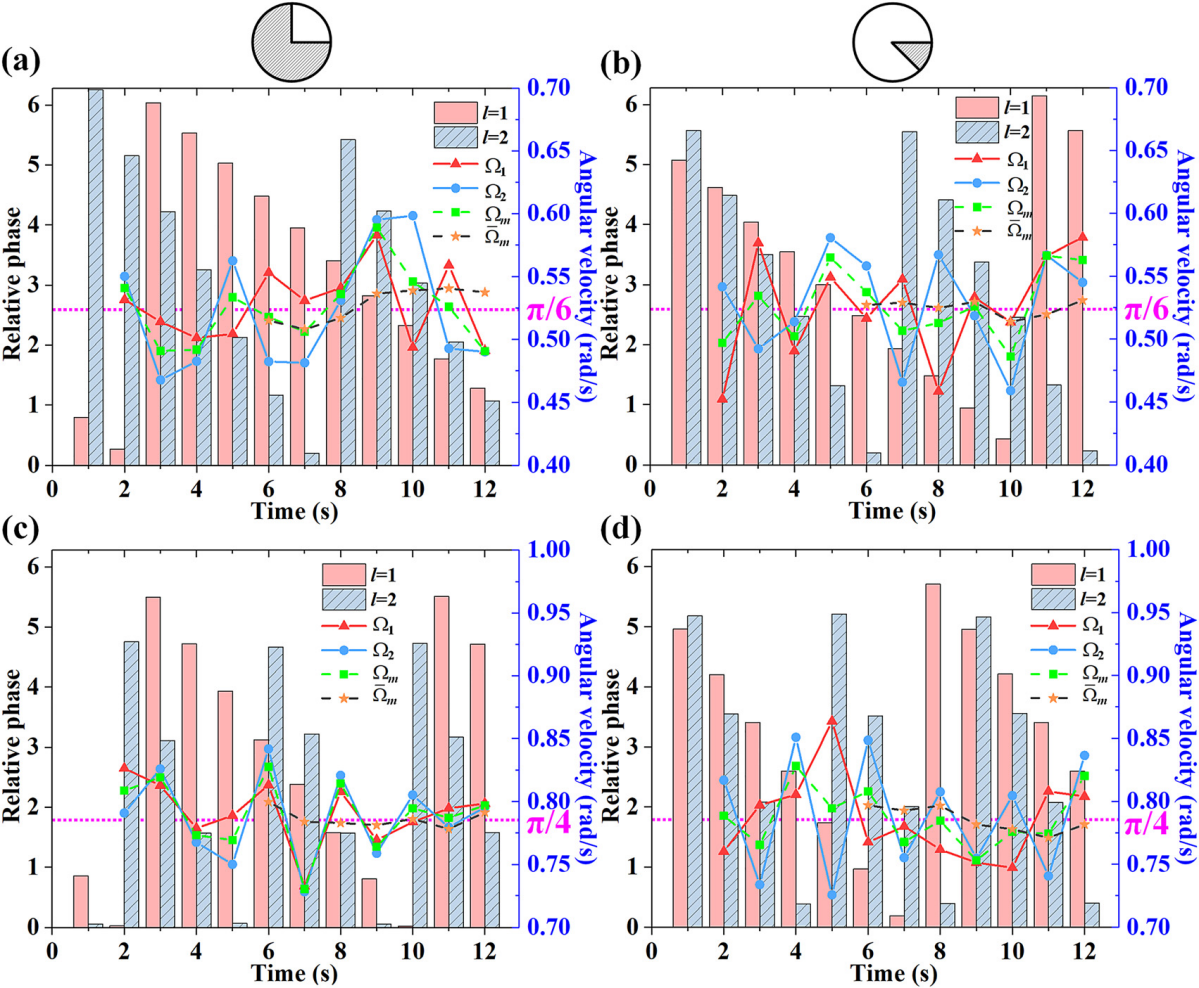
Detecting angular velocity using OAM phase spectra. (a) The opening angle and angular velocity of the object are *π*/2 and *π*/6 (rad/s), respectively. (b) The opening angle and angular velocity of the object are 7*π*/4 and *π*/6 (rad/s), respectively. (c) The opening angle and angular velocity of the object are *π*/2 and *π*/4 (rad/s), respectively. (d) The opening angle and angular velocity of the object are 7*π*/4 and *π*/4 (rad/s), respectively.

Based on the experimental measurements of the 2DPIMA, the energy of each focus and the OAM phase spectra were calculated instantaneously. Figure 5(a) and (b) show the OAM phase spectra obtained by 12 experimental measurements of the two spinning objects with an angular velocity *π*/6 (rad/s), respectively. While Figure 5(c) and (d) are the results when the angular velocity of the two objects increased to *π*/4 (rad/s). The pink histogram in Figure 5 indicates the phase of OAM state *l* = 1, while the cyan histogram represents the phase of OAM state *l* = 2. Then, the angular velocity is calculated with the phase variation of a single OAM state. The red triangle and blue circle in Figure 5 show the angular velocity of the object calculated by the single OAM state *l* = 1 and *l* = 2, respectively. According to Figure 5, we can see that the angular velocity measurements $\Omega_{1}$ and $\Omega_{2}$ fluctuate near the actual angular velocity, and the measurement error is large.

To reduce the measurement error, both OAM states *l* = 1 and *l* = 2 are employed simultaneously to measure the mean angular velocity $\Omega_{m}$ of the spinning object, which is marked by the green square in Figure 5. The maximum measurement error of the mean angular velocity is 12.47% in Figure 5(a), 8.20% in Figure 5(b), 7.20% in Figure 5(c), and 9.96% in Figure 5(d), which is still large. In order to avoid the contingency of a single measurement, we expand the number of measurement samples. As the hybrid phase plate used to generate the 2DPIMA is fixed, the highest frequency of the detection system only depends on the frequency of the CCD camera at the receiving end, which allows us to increase the sampling frequency by photodetectors and get more experimental data to reduce the measurement error. Therefore, the definition of weighted mean angular velocity ${\bar{\Omega }}_{m}$ is introduced, which is the average value of the latest five calculation results of the mean angular velocity $\Omega_{m}$ . As shown in Figure 5 with the yellow pentagram, before calculating the weighted mean angular velocity, the latest six measurements of 2DPIMA need to be stored first. After the sixth relative phase measurement, we can get five $\Omega_{m}$ and a further one ${\bar{\Omega }}_{m}$ . Then the weighted mean angular velocity is calculated with six measurements, and the average measurement error (the average of ${\bar{\Omega }}_{m}$ ) is sharply reduced to 2.45% for the first object and 1.01% for the second object (when the angular velocity of both objects is *π*/6 rad/s) and 1.82% for the first object and 1.81% for the second object (when the angular velocity of both objects is *π*/4 rad/s).

For comparison, we use the slope of the OAM phase spectrum (*l* = 1 and *l* = 2) to calculate the instantaneous azimuthal angle of the spinning object and further calculate the angular velocity by using azimuth angle variation (see Supplementary Figure S2). Because the calculated value of the slope is greatly affected by the phase measurement error, it is not feasible to use the relationship between the phases of different OAM modes to measure the angular velocity. When measuring the angular velocity of a spinning object using the phase variation of OAM states, the measurement error is sharply reduced. In addition, the experimental data also show that the method of measuring the angular velocity is not affected by the value of the opening angle of the slit. As shown in Eqs. (3) and (4), the opening angle *θ* of the spinning object does not affect the OAM phase variation measurement. Specifically, limited by the resolution of SLM, only two OAM modes were employed in this work. The measurement error can be further reduced by using more OAM states, which needs the help of a higher resolution SLM.

## 5 Conclusions

In summary, we proposed a phase-to-intensity strategy to measure the angular velocity of a spinning object using the OAM phase spectrum. The linear relationship between the OAM phase spectrum variation in the truncated beam and the angular velocity of the object was proved theoretically. In order to measure the variation of the OAM phase, we proposed a real-time multi-OAM phase measurement scheme, in which a 2DPIMA is generated and the relative phase of one OAM state can be calculated instantaneously with the intensities of four spots. By measuring the phase variation of OAM modes in a time, the rotational angular velocity of a spinning object can be calculated. The experimental results show that the proposed method has high accuracy with only several measurements. The OAM-based angular velocity detection, which demonstrates the feasibility of measuring the speed of moving objects by using the OAM phase spectrum, can be used as an effective supplement to the rotational Doppler effect for measuring low-speed spinning objects and maybe find potential applications in OAM-related remote sensing.

## Supplementary Material

Supplementary Material
