# Supplementary material for: A phase-to-intensity strategy of angular velocity measurement based on photonic orbital angular momentum
Source: Nanophotonics. 2021 Sep 28;11(4):865–72. doi: 10.1515/nanoph-2021-0461 (PMC11501140; doi:10.1515/nanoph-2021-0461)
Supplement: Supplementary file 1 — Supplementary Material [file j_nanoph-2021-0461_suppl.docx]

# Supplementary Information for

# A phase-to-intensity strategy of angular velocity measurement based on photonic orbital angular momentum

Duo Deng^1,2,3^, Hua Zhao^2^, Jincheng Ni^3^, Yan Li^1,^*, and Cheng-Wei Qiu^3,^*

^1^Department of Optoelectronics Science, Harbin Institute of Technology, Weihai 264209, China

^2^School of Physics, Harbin Institute of Technology, Harbin 150001, China

^3^Department of Electrical and Computer Engineering, National University of Singapore, Singapore 117583, Singapore

^*^To whom correspondence may be addressed. Email: liy@hit.edu.cn, eleqc@nus.edu.sg.

**This file includes:**

Supplementary Notes 1-4

Table S1

Figs. S1 and S2

**Supplementary Note 1 (The OAM intensity spectra in the truncated beam passing through a spinning object do not change** **with the rotation of the spinning object)**

When a Gaussian beam illuminates on an object with an angular aperture, the light beam is partially blocked by the object and only part of the light beam can pass through the object. The diffraction field of the truncated beam could be expressed as

 (S1)

where *l*_0_, *A*(*r*), and *φ*_0_ are the topological charge, amplitude, and phase of the incident OAM beam. *θ* and *ϕ*_0_ are the opening angle and azimuthal angle of the object’s slit. *r* and *ϕ* are the normalized polar coordinates on the receiving plane. *R* is the radius of the incident beam. *k*=2π/λ is the wavenumber, with λ is the vacuum wavelength. *z* is the propagation distance.

The angular restriction of light modifies the OAM spectrum of the incident beam, generating new OAM sidebands in the truncated beam, in which the complex amplitude of *l*-th OAM beam could be expressed as:

 (S2)

Therefore, the OAM intensity spectrum in the truncated beam could be evaluated to:

$\left|A_{l}\left(\theta\right)\right|^{2}=\left(A\right)^{2}\left(\frac{\theta}{2\pi}\right)^{2}\mathrm{sinc}^{2}\left[\frac{\theta}{2}\left(l_{0}-l\right)\right].$ (S3)

From Eq. (S3), we could see that the intensity of *l*-th OAM state in the truncated beam is closely related to the opening angle of the object. While the azimuthal angle *ϕ*_0_ doesn’t influence the intensity of OAM beam.

When the object is spinning, the diffraction field of the truncated beam could be rewritten as

 (S4)

The complex amplitude of *l*-th OAM beam in Eq. (S4) could be calculated as:

 (S5)

The OAM intensity spectrum in the truncated beam passing through spinning object could be expressed as:

$\left|A_{l}\left(\theta\right)\right|^{2}=\left(A\right)^{2}\left(\frac{\theta}{2\pi}\right)^{2}\mathrm{sinc}^{2}\left[\frac{\theta}{2}\left(l_{0}-l\right)\right].$ (S6)

which do not change with the rotation of the object.

**Supplementary Note 2 (Emulation of a spinning object with SLM)**

To emulate the spinning object with a phase-only spatial light modulator, a computer-generated hologram is uploaded on the SLM_1_. The transmission function of the computer-generated hologram is

 (S7)

The 0 transmission is realized with a fan-shaped blazed grating, which could deflect the corresponding region of the incident beam.

**Supplementary Note 3 (Optical apparatus)**

The Gaussian beam emitted by diode-pumped solid-state laser (MGL-III-532) with a central wavelength of 532 nm is spatially filtered using a single-mode fiber. The phase-only SLM (HDSLM63R) has 1280×720 pixels, with a pixel pitch of 6.3 μm, target surface size 8×4.54 mm, and a refresh rate of 60 Hz, on which a computer-generated hologram with 256 gray levels is displayed. The intensity pattern of the 2DIPMA is obtained on a CCD with 2592×1944 pixels and pixel pitch 2.2μm (frame rate 63fps@1024×768).

**Supplementary Note 4 (Details of the phase-to-intensity measurement)**

The two-dimensional phase-to-intensity measurement array is captured by a CCD (The commercial photodetectors can be placed in the appropriate positions to achieve fast instantaneous detection). The received pattern is converted into a 256-order gray image. At the preordained position of each focus, a 16×16 pixels square lattice is intercepted. Calculating the sum of each pixel’s gray value of the square lattice, the intensity of the focus is calculated. The sum of every pixel’s gray value of the spot received by the camera is proportional to the real intensity of the spot when the received power is lower than the camera’s threshold:

 (S8)

where *W*_n_ is the intensity of spot I_n_ measured by CCD, *P* is the scaling factor. As the four spots used to calculate the relative phase are placed in the same focal plane (The scaling factor is consistent when calculating their intensities), the phase-to-intensity measurement is not affected by the scaling factor:

 (S9)

When calculating the OAM phase with the intensities of the corresponding four spots, the focus energy measurement errors will lead to the error of OAM phase calculation, and further affect the accuracy of the OAM-based angular velocity detection. As shown in Fig. 5 with red triangle and blue circle, the angular velocity measurements based on single OAM state fluctuate near the actual angular velocity. Thus, to reduce the measurement error, 2 OAM state and 6 measurement samples are used to measure a weighted mean angular velocity.

Furthermore, there are some spots with small intensities in the 2DPIMA. These weak spots are more sensitive to the focus energy measurement error. It seems that the weak spots will lead to large phase measurement error. However, due to the *atan* function, it will not greatly affect the phase measurement. The reason is as follows:

$\mathrm{atan}\left(\frac{\text{I}_{S}-\text{I}_{W}}{\text{I}_{S}-\text{I}_{W}}\right)=\mathrm{atan}\left(\frac{\left(\text{I}_{S}^{1}+\text{I}_{e}^{1}\right)-\left(\text{I}_{W}^{3}+\text{I}_{e}^{3}\right)}{\left(\text{I}_{S}^{2}+\text{I}_{e}^{2}\right)-\left(\text{I}_{W}^{4}+\text{I}_{e}^{4}\right)}\right)\approx\mathrm{atan}\left(\frac{\text{I}_{S}^{1}}{\text{I}_{S}^{2}}\right)$ (S10a)

$\begin{matrix} \mathrm{atan}\left(\frac{\text{I}_{S}-\text{I}_{W}}{\text{I}_{W}-\text{I}_{W}}\right)=\mathrm{atan}\left(\frac{\left(\text{I}_{S}^{1}+\text{I}_{e}^{1}\right)-\left(\text{I}_{W}^{3}+\text{I}_{e}^{3}\right)}{\left(\text{I}_{W}^{2}+\text{I}_{e}^{2}\right)-\left(\text{I}_{W}^{4}+\text{I}_{e}^{4}\right)}\right)\approx\mathrm{atan}\left(\frac{\text{I}_{S}^{1}}{\text{I}_{W}^{2}-\text{I}_{W}^{4}+\text{I}_{e}^{2}-\text{I}_{e}^{4}}\right) \\ \text{ }\approx\mathrm{atan}\left(x\right)\left(x\to\infty\right) \end{matrix}$ (S10b)

$\begin{matrix} \mathrm{atan}\left(\frac{\text{I}_{W}-\text{I}_{W}}{\text{I}_{S}-\text{I}_{W}}\right)=\mathrm{atan}\left(\frac{\left(\text{I}_{W}^{1}+\text{I}_{e}^{1}\right)-\left(\text{I}_{W}^{3}+\text{I}_{e}^{3}\right)}{\left(\text{I}_{S}^{2}+\text{I}_{e}^{2}\right)-\left(\text{I}_{W}^{4}+\text{I}_{e}^{4}\right)}\right)\approx\mathrm{atan}\left(\frac{\text{I}_{W}^{1}-\text{I}_{W}^{3}+\text{I}_{e}^{1}-\text{I}_{e}^{3}}{\text{I}_{S}^{2}}\right) \\ \text{ }\approx\mathrm{atan}\left(x\right)\left(x\to0\right), \end{matrix}$ (S10c)

where I*_S_*, I*_W_*, and I*_e_* represent the intensity of strong spot, weak spot, and the focus energy measurement error (I*_S_* >> I*_W_*, I*_e_*). When there are both strong spots and weak spots in a numerator or a denominator, as shown in Eq. (S10a), the phase measurement has little relationship with weak spots. When two weak spots are present in a numerator or a denominator, as shown in Eqs. (S10b) and (S10c), the arguments in the *atan* function will approach zero or infinity, which also have little impact on the phase measurement.

**Table S1. Some typical works of measuring angular velocity of spinning objects using rotational Doppler effect**

| Item | Object’s angular velocity (rad/s) | The OAM state(s) of the probe beam |
| --- | --- | --- |
| Science, 341: 537, 2013 | 383 | ±18 |
| Optica, 1: 1, 2014 | 243 | ±12 |
| Opt. Lett., 40: 5778, 2015 | ~10^8^ | ±1 |
| Opt. Lett., 41: 2549, 2016 | 50π | 1 |
| Opt. Lasers. Eng., 124: 105842, 2020 | 200-600 | ±16 |
| Opt. Express, 29:4058, 2021 | 257.8 | ±4 |

*The proposed OAM-based angular velocity detection can measure spinning objects at π/6 (rad/s) by using the phase variation of OAM beams *l*=1 and *l*=2.


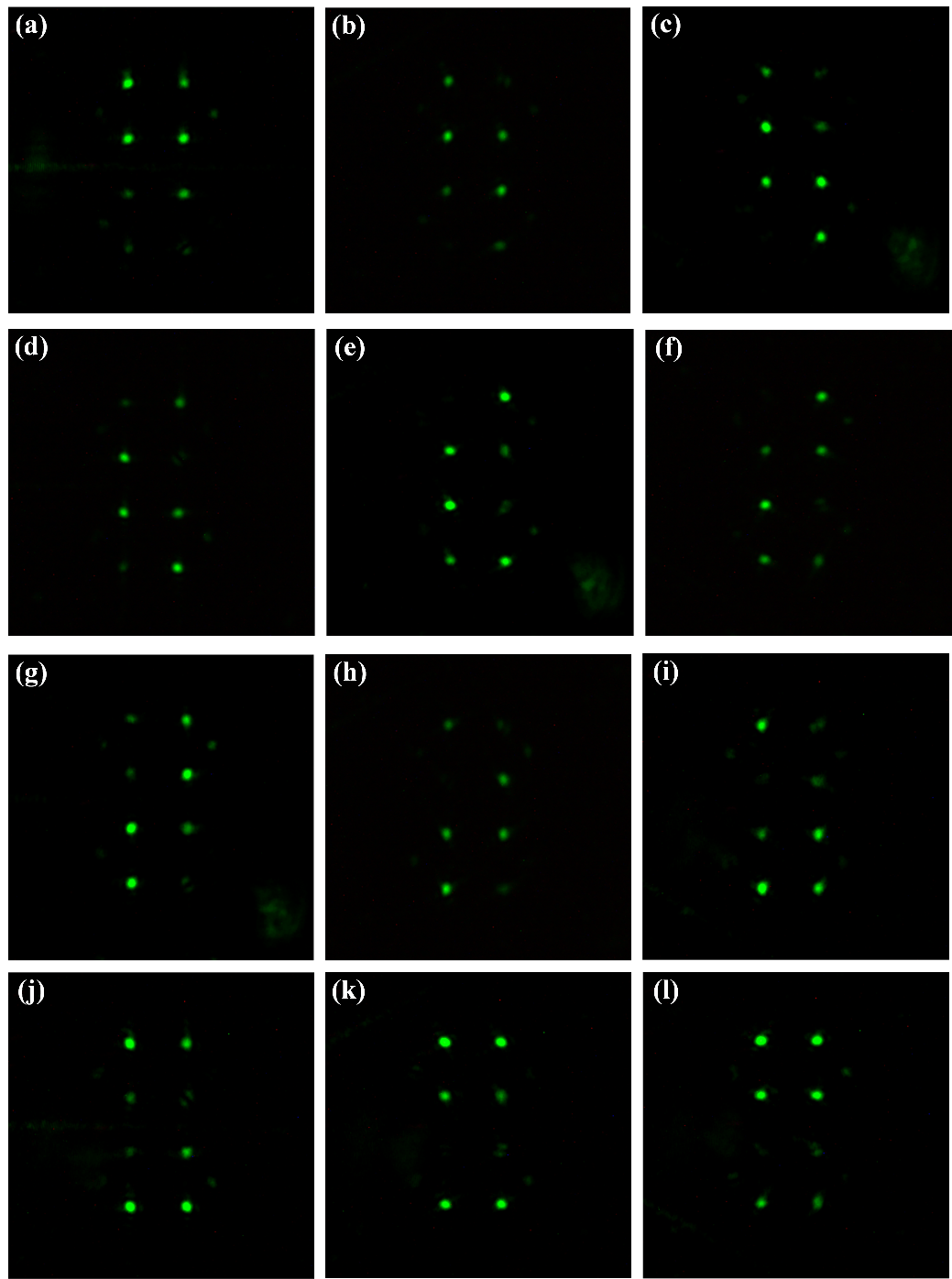


**Fig. S1.** **Experimental results of the object whose opening angle and angular velocity are** **π/2 and π/6 (rad/s).** Measured at (a) *t*_0_, (b) *t*_0_+1s, (c) *t*_0_+2s, (d) *t*_0_+3s, (e) *t*_0_+4s, (f) *t*_0_+5s, (g) *t*_0_+6s, (h) *t*_0_+7s, (i) *t*_0_+8s, (j) *t*_0_+9s, (k) *t*_0_+10s, (l) *t*_0_+11s.


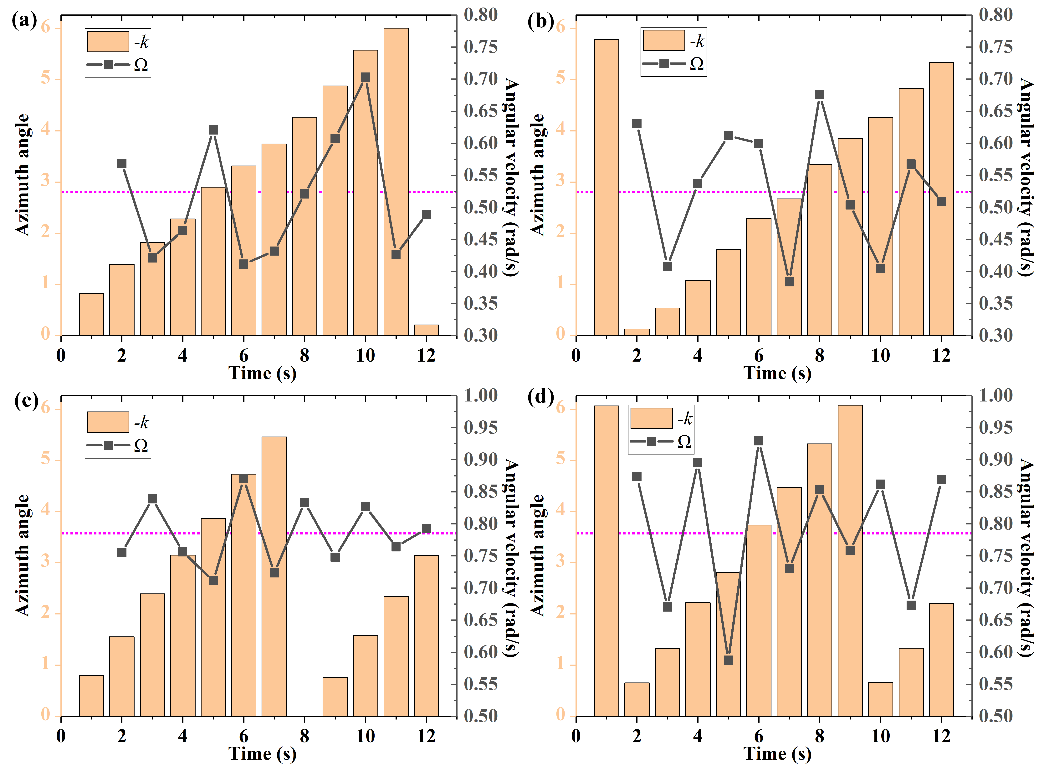


**Fig. S2.** **Calculating angular velocities of spinning objects with the slope of OAM phase spectrum.** The opening angle and angular velocity of the objects are (a) π/2 and π/6 (rad/s), (b) 7π/4 and π/6 (rad/s), (c) π/2 and π/4 (rad/s), (d) 7π/4 and π/4 (rad/s). The slope is calculated with the phases of OAM state *l*=1 and *l*=2, which are determined with the 2DPIMA as shown in Fig. 5. The azimuth angle of the object is calculated with the slope of OAM phase spectrum and the angular velocity could be calculated with the azimuth angle variation of the object. The maximum measurement error reaches (a) 34.29%, (b) 29.07%, (c) 10.86%, (d) 25.17%.
